# The Effects of Select Hydrocolloids on the Processing of Pâté-Style Canned Pet Food

**DOI:** 10.3390/foods10102506

**Published:** 2021-10-19

**Authors:** Amanda N. Dainton, Hulya Dogan, Charles Gregory Aldrich

**Affiliations:** Department of Grain Science & Industry, Kansas State University, 201 Shellenberger Hall, 1301 Mid Campus Drive, Manhattan, KS 66506, USA; adainton@ksu.edu (A.N.D.); dogan@ksu.edu (H.D.)

**Keywords:** expressible moisture, gel, gum, heat penetration, thermally processed, texture, wet pet food

## Abstract

Hydrocolloids are commonly used in canned pet food. However, their functional effects have not been quantified in this food format. The objective was to determine the effects of select hydrocolloids on batter consistency, heat penetration, and texture of canned pet food. Treatments were added to the formula as 1% dextrose (D) and 0.5% guar gum with 0.5% of either dextrose (DG), kappa carrageenan (KCG), locust bean gum (LBG), or xanthan gum (XGG). Data were analyzed as a 1-way ANOVA with batch as a random effect and separated by Fisher’s LSD at *p* < 0.05. Batter consistency (distance traveled in 30 s) thickened with increasing levels of hydrocolloids (thinnest to thickest: 23.63 to 2.75 cm). The D treatment (12.08 min) accumulated greater lethality during the heating cycle compared to all others (average 9.09 min). The KCG treatment (27.00 N) was the firmest and D and DG (average 8.75 N) the softest with LBG and XGG (average 15.59 N) intermediate. Toughness was similar except D (67 N·mm) was less tough than DG (117 N·mm). The D treatment showed the greatest expressible moisture (49.91%), LBG and XGG the lowest (average 16.54%), and DG and KCG intermediate (average 25.26%). Hydrocolloids influenced heat penetration, likely due to differences in batter consistency, and affected finished product texture.

## 1. Introduction

Canned pet foods are commercially sterilized, low-acid products and come in formats similar to stews and pâtés in appearance. These products primarily consist of meats and water and contain binding/structural ingredient systems similar to restructured meat products for human consumption [1,2]. Hydrocolloids, such as carrageenan and guar, locust bean, and xanthan gums, are common choices. They are able to increase the viscosity of unprocessed meat batters and emulsions [3,4] and may increase or decrease the firmness of a finished product, depending on the formulation [5,6]. These differences in functionality are driven by their chemical structures. Briefly, kappa carrageenan is a linear molecule with repeating galactose units and 3,6-anhydrogalactose units connected with alternating α-1,3 and β-1,4 bonds [2]. Xanthan gum consists of a 1,4 linked β-d-glucose backbone with a side chain of a glucuronic acid and two mannose units every other glucose unit. Locust bean gum and guar gum are the most similar as they have the same linear 1,4-β-d-mannose backbone with galactose side chains connected with 1,6-α-glycosidic bonds. However, they differ in their galactose content; locust bean gum contains less galactose by weight compared to guar gum (17–26% vs. 33–40%) [2]. Though it is likely that these ingredients are included in commercial pet foods for similar functional benefits, this has not been a primary area of investigation. Instead, research has focused on the nutritional value of carbohydrate hydrocolloids as soluble fibers [7,8,9].

The incorporation of hydrocolloids in commercial pet foods has waned as companies wish to differentiate their products from their competitors’ offerings [10]. There are no reports of why pet food companies use the inclusion of hydrocolloids or lack thereof to distinguish their formulas from others. Mixed findings suggest that the addition of similar ingredients to canned pet foods causes softer stools in dogs [11] while others reported firmer stools compared to a control diet [12]. A small segment of the population believes some hydrocolloids to be toxic or carcinogenic to dogs and cats, though this is not confirmed in the literature [13,14]. Regardless, pet food companies are looking for new ingredients to replace the commonly used hydrocolloids. However, there are no reports quantifying and differentiating the functional effects of these ingredients in canned pet foods. This limits the ability to identify alternative, label friendly ingredients with similar functionality. Research with sausage, meatballs, and other restructured meat products can provide some insight, but many of these products utilize hydrocolloids for their ability to mimic the mouthfeel of fat [2]. As such, conclusions from restructured meat products for human consumption may not be directly applicable due to differences in formulation and processing methods.

The objective of this experiment was to characterize the physicochemical and processing effects of guar gum and blends of guar gum with another hydrocolloid on the processing of canned pet foods. The hypothesis was that the addition of hydrocolloids would decrease heat penetration and alter the color and texture of processed foods. Additionally, systems containing guar gum with an additional hydrocolloid would have measurable differences in texture driven by the mechanism of the additional hydrocolloid.

## 2. Materials and Methods

### 2.1. Formulation of Canned Pet Foods

Five treatments were designed to show the effects of guar gum, kappa carrageenan, locust bean gum, and xanthan gum on functional properties of canned pet food. Dextrose was chosen as a space-holding control ingredient for its aqueous solubility [15] and its similar moisture content compared to the carbohydrate hydrocolloids of interest. Controlling the moisture content of the samples was a concern to minimize confounding effects on heat penetration [16]. Guar gum was specifically chosen because of its high thickening power [2] and its prevalence in commercial canned pet foods. As such, guar gum was also included in treatments containing either kappa carrageenan, locust bean gum, and xanthan gum in an attempt to mimic commercial pet foods.

There are limited recommendations for the inclusion level of these ingredients in canned pet food. Locust bean gum is used in pet foods at 0.2–0.5% [2] for its binding effects, or the ability to increase interactions between macromolecules themselves and with the solvent [17]. Additionally, a model lunch meat formula contained 0.6% kappa carrageenan for its gelling effects [2]. The inclusion level of 0.5% for individual hydrocolloids was chosen based on these recommendations as well as preliminary data wherein higher inclusion levels were firmer than commercial canned pet foods (data not presented).

This led to the creation of five experimental treatments (Table 1): 1% dextrose (D), 0.5% dextrose and 0.5% guar gum (DG), 0.5% guar gum and 0.5% kappa carrageenan (KCG), 0.5% guar gum and 0.5% locust bean gum (LBG), and 0.5% guar gum and 0.5% xanthan gum (XGG).

Prior to diet production, frozen blocks of mechanically deboned chicken (CJ Foods, Bern, KS, USA) were ground with a lab-scale meat grinder (Weston Pro Series #32, Southern Pine, NC, USA) fitted with a die plate with 7 mm diameter holes. Treatments were replicated three times over three days of production, with each treatment made on each day. Water was heated in a stock pot until it reached 40 °C, at which point the tempered ground chicken was added and the mixture was brought back up to 40 °C. Brewer’s rice (Lortscher Animal Nutrition, Bern, KS, USA), spray-dried egg white (Rembrandt Foods, Okoboji, IA), sunflower oil (Kroger, Manhattan, KS, USA), potassium chloride (Lortscher Animal Nutrition, Bern, KS, USA), vitamin premix (Lortscher Animal Nutrition, Bern, KS, USA), and trace mineral premix (Lortscher Animal Nutrition, Bern, KS, USA) were added to the stock pot and heated to 60 °C with continuous stirring. Once the batter reached the target temperature, dextrose (Fairview Mills, Seneca, KS, USA) and/or the respective hydrocolloid ingredient(s) (Danisco, New Century, KS, USA) were added and the batter was stirred continuously for 5 min while maintaining temperature. Treatment order was randomized each day to maintain similar initial internal can temperatures, which can influence heat penetration and the required length of processing [18,19]. After mixing, 21 cans (size 300 × 407; House of Cans, Lincolnwood, IL, USA) were filled with 405 ± 5 g of batter for each treatment.

### 2.2. Analysis of Pre-Thermal Processing Batters of Canned Pet Food

Three consistency measurements were taken per treatment replication using a Bostwick consistometer (CSC Scientific Company, Fairfax, VA, USA). Briefly, this analysis utilized a sloped trough and slide gate to determine how thick or thin a sample of a set volume was. Measurements of distance in centimeters traveled in 30 s were recorded. A sample that traveled farther was considered to have thinner consistency and a sample that did not travel as far was considered to have thicker consistency. The Bostwick consistometer methodology was chosen because it does not require room temperature samples, which is a concern for viscosity analysis. Generally, viscosity of food samples is greater (i.e., thicker) at cooler temperatures [3] and those values would not be relevant for samples collected directly from production. In the present experiment, all samples were analyzed at the same temperature immediately after the complete batter was mixed for 5 min at 60 °C. Additionally, the Bostwick consistometer is widely used by the pet food industry because of its low cost and limited required training [20,21].

Three pH measurements were taken with a pH meter (P/N 54X002608; Oakton Instruments, Vernon Hills, IL, USA) fitted with a pointed pH probe (model #FC240B; Hanna Instruments, Smithfield, RI, USA). Finally, three samples for water activity were collected and stored in covered containers to return to room temperature for measurement with a water activity meter (Decagon CX-2; Meter Group, Pullman, WA, USA).

### 2.3. Analysis of Processing Control Measures and Thermal Processing Calculations

Treatments prepared on the same day were processed in the retort at the same time. Four thermocouples (Ecklund-Harrison Technologies, Fort Meyers, FL) per treatment were placed in cans prior to filling and connected to a data capture system (CALSoft v. 5; TechniCAL LLC, Metairie, LA, USA) to record temperature in the center of the cans during processing. Fill weight and gross headspace were recorded for these cans as well. Specifically, gross headspace was measured as the distance from the top of the can body to the top of the batter inside the container. Once measurements were taken, lids (size 300 × 407 sanitary lids; House of Cans, Lincolnwood, IL, USA) were sealed onto the cans with a seamer (Dixie Seamer, 91118; Athens, GA, USA). Cans were randomly loaded into a still retort (Dixie, 00-43; Athens, GA, USA) and processed at 144.79 kPa and 121 °C. Thermocouple-containing cans were randomly distributed among all other cans. Temperature inside the retort was also recorded by the data capture system. The intent was for the data capture system to record temperature inside the retort and inside each can every 15 s. However, the data capture system could not consistently record temperature at this rate during production days 1 and 2. The longest length of time between temperature measurements was 7.75 min and the average ± standard deviation excluding the normal time intervals was 1.23 ± 1.30 min. The cooling cycle was started once the coldest can among all treatments in the retort containing a thermocouple achieved a minimum lethality, or the relative amount of time at a constant reference temperature of 121.11 °C [22], of 8 min. This value has been reported as a minimum for commercial canned pet food [23] and was chosen to remain consistent with pet food industry practices. Cans were cooled in the retort with municipal water (20 °C) until the last can containing a thermocouple dropped below 50 °C before removal from the retort.

Calculations for lethality (Equation (1)) [22] and cook value (C_100_; Equation (2)) were made using the thermocouple temperature data (Figure S1). T_C_(t) is the internal can temperature at any given time t and Δt represents the length of time between temperature measurements. The reference temperature and the z-value representing the change in temperature required to see a 1 log reduction in the D value, or the amount of time required to see a 1 log reduction, were specific to the item of interest [22,24]. These values were 121.11 and 10 °C, respectively, for the calculation of lethality. The z value came from experiments measuring the heat resistance of *Clostridium botulinum* 213-B in a pH7 phosphate buffer [25] and had been used in a preliminary experiment with thermally processed pet food [26]. The C_100_ calculation utilized the reference temperature (100 °C) and z value (33 °C) for thiamin, the weakest nutrient. Both equation integrals were solved using the trapezoid rule (Equations (3) and (4), respectively). These calculations were used to discuss the effect of the treatments on heat penetration and dissipation. For example, higher lethality and C_100_ during the heating retort cycle were indirect indicators of faster heat penetration rates. This methodology was used in a preliminary study of the effects of container size and type on lethality values of wet pet food processed for the same amount of time [26].

(1) $$Lethality=\int_{0}^{t}10^{\frac{T_{C}\left(t\right)-121.11{}^{\circ}C}{10{}^{\circ}C}}\Delta t,$$

(2) $$C_{100}=\int_{0}^{t}10^{\frac{T_{C}\left(t\right)-100{}^{\circ}C}{33{}^{\circ}C}}\Delta t,$$

(3) $$Lethality=\overset{t}{\underset{0}{\sum }}10^{\frac{T_{C}\left(t\right)-121.11{}^{\circ}C}{10}}\Delta t,$$

(4) $$C_{100}=\overset{t}{\underset{0}{\sum }}10^{\frac{T_{C}\left(t\right)-100{}^{\circ}C}{33}}\Delta t,$$

### 2.4. Analysis of Processed Canned Pet Foods

Four cans per combination of treatment and production day were blended (14-speed Osterizer; Sunbeam Products, Boca Raton, FL, USA) and freeze dried (model #HR7000-L; Harvest Right, LLC, Salt Lake City, UT, USA) for analysis of moisture content (AOAC 934.01) in duplicate. Can vacuum was measured on 4 cans per treatment replicate with a glycerin-filled vacuum gauge (#25.300/30; Fisher Scientific, Hampton, NH USA) fitted with a rubber collar (#10816-11; Wilkens-Anderson Co., Chicago, IL, USA) and metal tip.

Three cans per treatment replicate were analyzed for pH (meter: P/N 54X002608, Oakton Instruments, Vernon Hills, IL; probe: model #FC240B, Hanna Instruments, Smithfield, RI, USA), free liquid, and expressible moisture by centrifugation as an indication of water holding capacity [27]. Briefly, free liquid was quantified as the mass of the liquid phase, if present, upon opening the can. Expressible moisture was determined by weighing approximately 1 g of sample into two Whatman Grade 3 filter papers (GE Healthcare Life Sciences, Piscataway, NJ, USA) and one Whatman Grade 50 filter paper (GE Healthcare Life Sciences, Piscataway, NJ, USA) folded into a thimble shape and centrifuging in a 50 mL polypropylene centrifuge tube (Globe Scientific Inc., Mahwah, NJ, USA) at 2000× *g* (Sorvall^®^ RC 6 Plus; Thermo Electron Corporation, Waltham, MA, USA). As-is and dried filter paper weights were recorded before and after centrifuging to account for residue transfer to the filter papers [Equations (5)–(7)]. Analysis of expressible moisture was conducted in quadruplicate for each can per treatment replicate.

(5) Expressed moisture+residue, g=As−is filter paper after centrifugation, g−As−is initial filter paper, g,

(6) $$\begin{array}{cc} Expressed\;residue,g & \\ = & Dried\;filter\;paper\;after\;centrifugation,\;g\\ - & Dried\;initial\;filter\;paper,\;g, \end{array}$$

(7) $$Expressible\;moisture,\;\%=\frac{Equation\;\left(5\right)-Equation\;\left(6\right)}{As-is\;weight\;of\;sample}*100,$$

Texture was characterized with a modified back extrusion test using a texture analyzer (TA-XT2; Texture Technologies Corp., Hamilton, MA, USA) fitted with a 5.08 cm diameter and 2 cm tall cylindrical probe and a 30 kg load cell. The trigger force was set to 5 g and the test speeds (pre-, test, and post-) were set to 1 mm/s. The probe was pressed into the center of the products in cans to a depth of 2 cm. Firmness was recorded as the largest force measurement observed during the 2 cm compression. Often this value was similar to or not different from the force measurement recorded at the end of the compression. Toughness was calculated as the area under the curve of the compression peak using the trapezoid rule (Figure S2). Five cans from each treatment replicate were analyzed and values were averaged together to generate composite values for each replicate. Cans were selected from the beginning, middle, and end of the filling sequence for each treatment to accurately capture texture for the entire production. This methodology was selected instead of a texture profile analysis procedure because some of the treatments did not form structures that would remain stable if removed from the can. A similar methodology was applied to canned cat foods processed to comparable lethality values with different processing conditions [28].

Three cans per replicate of treatment were analyzed for color with a CIELAB color-space colorimeter (CR-410 Chroma Meter, Konica Minolta, Chiyoda, Tokyo, Japan) with five measurements taken from four evenly spaced regions of pâté from each can. Sections were created by removing the product from the can and slicing 3 times with a knife. In cases where slices could not be created, color of the top and bottom of the product were measured while inside the can and internal color by separating product into 3 sections of roughly the same size. Color was described in terms of L* (brightness), a* (red to green scale), and b* (yellow to blue scale). Values of L* closer to 100 indicated lighter products, whereas values of L* closer to 0 indicated darker products. The red to green and yellow to blue scales contained negative and positive values. More negative values of a* and b* indicated greener and bluer color, respectively. On the other hand, more positive values of a* and b* indicated redder and yellower color, respectively. All three scales form a three-dimensional space with the intersection point in the middle of each scale.

### 2.5. Statistical Analysis

Data were analyzed as a randomized complete block design with treatment as the fixed effect and day as the random block using statistical analysis software (SAS 9.4; SAS Institute, Cary, NC, USA). Values were presented as least square means ± the standard error of the mean and differences were calculated using Fisher’s LSD in the GLIMMIX procedure. The CORR procedure was used to calculate r and *p*-values for Pearson correlations. All tests were considered significant if *p* < 0.05.

## 3. Results

### 3.1. Pre-Thermal Processing Batter Analyses

Batter pH and batter water activity were not different (*p* > 0.05) among treatments and averaged 5.94 and 0.990, respectively (Table 2). Consistency was affected (*p* < 0.05) by treatment. The D treatment was the thinnest (23.64 cm) and often traveled the full 24 cm in less than 30 s. Guar gum alone (DG = 6.60 cm) decreased (*p* < 0.05) consistency, resulting in a thicker batter, in comparison to D. The KCG, LBG, and XGG batters exhibited the lowest consistency with no differences (*p* > 0.05) between them (average = 2.75 cm).

### 3.2. Processing Control Analysis and Thermal Processing Values

Data from three thermocouples were removed from statistical analysis due to failure (2) and low fill weight (1). No differences (*p* > 0.05) were noted in can fill weight (average = 406.6 g), gross headspace (average = 13.87 mm), initial internal can temperature (average = 55.67 °C), or post-processing can vacuum (average = −12.9 kPa) across treatments (Table 3).

Heating cycle length and cooling cycle length across the three days averaged 81.25 ± 1.392 and 79.58 ± 22.735 min, respectively. The total, heating, and cooling lethalities were all affected (*p* < 0.05) by the treatments (Table 4). Heating lethality was greater (*p* < 0.05) for D (12.08 min) compared to the four other treatments (average = 9.09 min). The same relationship was observed for total lethality (D = 20.24 min; average of all others = 18.46 min). On the other hand, D (8.17 min) accumulated lower (*p* < 0.05) cooling lethality than LBG and XGG (average = 9.60 min) with DG and KCG not different (*p* > 0.05; average = 8.97 min) from any of the treatments. The total C_100_ was not affected (*p* > 0.05; average = 197.10 min) by the treatments. However, D accumulated more (*p* < 0.05) C_100_ during the heating cycle (137.01 min) and less (*p* < 0.05) C_100_ during the cooling cycle (64.90 min) compared to all other treatments (averages = 117.24 and 78.66 min, respectively). Total lethality and C_100_ were very strongly correlated (r = 0.98; *p* < 0.0001). The same relationship was observed between heating lethality and C_100_ (r = 1.00; *p* < 0.0001) and between cooling lethality and C_100_ (r = 0.95; *p* < 0.0001).

### 3.3. Physicochemical Quality of Processed Canned Cat Food

Many processed treatment characteristics were affected by the differences in carbohydrate hydrocolloid content (Table 5). The only experimental treatment to exhibit two phases was D, with 16.91% ± 1.629% of the product mass as a free liquid phase. The finished product pH was greatest (*p* < 0.05) for KCG (6.38) and lowest for dextrose-containing treatments (D and DG; average = 5.96) with LBG and XGG (average = 6.26) intermediate. Total moisture was greater (*p* < 0.05) for D and DG (average = 79.21%) than for LBG and XGG (average = 77.29%) with KCG (77.83%) intermediate and not different (*p* > 0.05) from any other treatment. Expressible moisture as a percentage of the total sample mass was greatest (*p* < 0.05) for D (49.91%) and lowest for LBG and XGG (average = 16.54%) with DG and KCG intermediate (average = 25.26%). The KCG treatment exhibited the greatest (*p* < 0.05) firmness and toughness (27.00 N and 370 N·mm, respectively) of all experimental treatments, followed by LBG and XGG (average = 15.59 N and 235 N·mm, respectively). The replacement of 0.5% dextrose with guar gum nearly doubled (*p* < 0.05) toughness (D = 67 N·mm; DG = 117 N·mm) but did not affect (*p* > 0.05) firmness (average = 8.75 N). Increasing the level of dextrose darkened (*p* < 0.05) the product. Additionally, LBG was lighter (*p* < 0.05) than DG with KCG and XGG intermediate and not different (*p* > 0.05). No differences (*p* > 0.05) in a* and b* were noted between KCG, LBG, and XGG (averages = 4.41 and 15.39, respectively). However, the inclusion of dextrose in D and DG resulted in redder (*p* < 0.05; average = 8.37) and yellower (*p* < 0.05; average = 22.05) color.

## 4. Discussion

The aim of this experiment was to quantify the functional characteristics present in canned pet food containing select hydrocolloids, specifically guar gum, kappa carrageenan, locust bean gum, and xanthan gum. Treatments were designed to show the effects of common hydrocolloid systems and guar gum alone at inclusion levels mimicking commercial canned pet food.

### 4.1. Characteristics of Pre-Thermal Processing Batters of Canned Pet Food

Consistency was affected by the treatments in the present experiment and generally thickened when the total hydrocolloid content of the treatment increased. Thickness of a hydrocolloid solution is dependent in the interactions between the hydrocolloid molecules and the solvent or liquid component of the system [17]. As such, increasing the amount of carbohydrate hydrocolloids increased the number of reactions possible with the solvent. The DG was approximately 3.5 times thicker than D, while KCG, LBG, and XGG were only an average of 2.4 times thicker than DG. Guar gum has a high thickening power compared to many carbohydrate hydrocolloids [2] because it contains many hydroxyl groups that form hydrogen bonds with water. There are no published reference values for consistency of pre-thermal processing batters of canned pet food. A batter with thinner consistency may be easier to mix and pump to the container filling station in a commercial pet food facility. However, a thinner consistency batter may splash more when containers are filled. This would contaminate the seam area, prevent a proper hermetic seal from forming, and expose the pet food to potential external contamination during and after thermal processing [29]. As such, consistency of pre-thermal processing batters should be considered when formulating new commercial pet foods.

Consistency was chosen as the metric to describe viscosity, which is known to affect the rate of heat penetration and the time required to reach lethality in food products [30]. The Bostwick consistometer does not measure viscosity directly and is influenced by other factors including gravitational forces, though it does allow for analysis of samples during can filling. Consistency is also listed in U.S. federal regulations as a potential critical factor for scheduled processes for thermally processed low-acid foods [31]. Studies with other food products have found conflicting results regarding the correlation between direct viscosity and Bostwick consistency measurements [32,33]. Similar research should be conducted with pet foods to validate the Bostwick consistometer as a method for apparent viscosity analysis.

Batter pH and batter water activity were not different among the treatments. The lack of difference in batter pH suggested that minimal reactions occurred during the 5 min mixing after the addition of hydrocolloids. It is also possible that differences may have been detected if the data were analyzed as the concentration of hydrogen ions instead of as pH values (i.e., the negative logarithm of the concentration of pH values). This would have yielded a range of average concentration of hydrogen ions from 1.07·10^−6^–1.25·10^−6^ hydrogen ions in the batters. The lack of difference observed in batter water activity was mainly influenced by the low concentration of hydrocolloids. Generally, carbohydrate hydrocolloids do not affect water activity when their inclusion level is less than 2% [1].

### 4.2. Thermal Processing Controls and Characteristics of Canned Pet Food

The intention of this experiment was to begin the cooling cycle after the last can containing a thermocouple reached a lethality value of 8 min. However, treatments appear slightly over-processed as the lowest average heating lethality was 8.69 min (Table 3). Similarly, another experiment struggled to achieve their targets when processing canned foods to different F_0_ values [23]. Three thermocouples failed during the present experiment, but more than the minimum 10 thermocouples recommended by the Institute for Thermal Processing Specialists [34] were successful across the three replicates for each treatment. Nevertheless, thermal processing parameters of initial internal can temperature, fill weight, gross headspace, and post-processing vacuum were constant. This indicated that differences in lethality were due to the treatments and not influenced by confounding factors. There are no published reference values for these parameters for commercial canned pet food. However, a preliminary study of canned pet food with initial internal can temperatures around 30 °C and can volumes of 88.7 and 162.7 mL observed post-processing can vacuums of −0.8 kPa [26]. Initial internal can temperatures in that experiment were roughly 50% colder than the present experiment and likely influenced the differences in post-processing can vacuum.

Differences were observed in the heating and cooling of the experimental treatments. Specifically, D obtained greater lethality and C_100_ during the heating phase and lower values during the cooling phase of retort processing. This could indicate a faster rate of heat penetration and heat dissipation compared to all other treatments. The thickening of pre-thermal processing batters due to the increase in hydrocolloid content likely increased the resistance to heat, leading to lower lethality and C_100_ when the foods were processed under the same time and temperature conditions. Previous research of the effect of viscosity on heat penetration found that increased food thickness decreased the average heating slope and increased the amount of time required to thermally process food [35]. This suggests that thinner food consistencies may benefit production facilities by decreasing the amount of time to process a food product, which could allow for more products to be made in the same amount of time. It is likely that the heating and cooking lag factors (j_h_ and j_c_, respectively) and the heating and cooling penetration factors (f_h_ and f_c_, respectively) were influenced by the treatments. The lag factors describe how long a food product initially takes to begin heating or cooling while the penetration factors describe the rate of heating or cooling [25]. The present experiment did not investigate these parameters, however, future experiments should do so to provide more understanding of how hydrocolloids affect thermal processing.

The C_100_ calculation has never been applied in literature to canned pet foods. This metric can describe the detrimental effect of increased thermal processing on quality changes such as texture and nutrients. Thiamin degradation is an important concern for pet foods, especially those for cats. Consumption of a thiamin deficient diet can be deadly within a few weeks [36,37]. Deficient pet foods should be recalled to prevent illness and death but recalls are costly to pet food companies. As such, this is a great concern for the pet food industry. However, there are no published reference values for acceptable and unacceptable cook values as it relates to thiamin content or other quality factors of canned pet food. The data presented in this study suggest that canned pet foods processed under commercial conditions have cook values of at least 195.91–201.90 min. Future experimentation needs to determine a maximum C_100_ before texture and thiamin are degraded to unacceptable levels.

### 4.3. Physicochemical Quality of Processed Canned Pet Food

Color was largely similar between KCG, LBG, and XGG, which was expected. Hydrocolloids are rarely involved in browning reactions. For example, an experiment with chicken sausages found that the level of carbohydrate hydrocolloids only explained 26.5% of the variation in lightness, 6.6% of the variation in redness, and none of the variation in yellowness [5]. Instead, other factors, such as fat inclusion level, were more influential. Differences in color were identified in pâté-style canned pet foods containing different soluble proteins at a 2.5% inclusion level [38]. Companies wishing to alter the color of their products with ingredients at low inclusion levels may have more success changing soluble proteins than carbohydrate hydrocolloids. Regardless, values for the lightness, redness, and yellowness of canned pet foods containing different carbohydrate hydrocolloids have never been published. A pilot study with canned pet foods presented CIELAB color-space values for commercial products, but the ingredient compositions were not disclosed [39]. As such, the values presented for KCG, LBG, and XGG could serve as reference values for chicken-based canned pet foods containing the respective hydrocolloids.

The D and DG treatments appear to have confounding factors influencing their color. First, D was processed to a higher total lethality, which increased the redness and yellowness of thermally processed shrimp in curry [40]. The DG treatment was similar to D in redness and yellowness, which suggested that degree of processing is not the only confounding factor. It is highly likely that the dextrose in both treatments participated in Maillard reactions during thermal processing. This reaction occurs between α-amino groups in proteins and reducing sugars [41] and is associated with increased redness and yellowness and more acidic pH levels in infant formula [42]. This suggested that D and DG could not serve as controls for redness and yellowness in the present experiment. There are no published values for the redness and yellowness of canned pet foods containing dextrose. The data for these two treatments are useful benchmarks for pet food companies who wish to use dextrose to increase the redness and yellowness of their products.

The pH levels of the processed foods were affected by the treatments. The D and DG treatments had more acidic pH, which could be tied to the production of Maillard reaction products mentioned in the previous paragraph. It also appeared that pH became more basic after thermal processing with the degree of change dependent on the carbohydrate hydrocolloids present. This would suggest that thermal processing caused a degree of hydrogen bonding, thus decreasing the amount of free hydrogen ions and explaining the shift to a more neutral pH for treatments LBG and XGG. The KCG treatment shifted even more because the sulfate half-ester groups in kappa carrageenan are negatively charged [1] and shift the pH even more. It may also be that the differences in pH are related to the differences in color. Chicken breasts classified by visual color assessment were further differentiated by pH and CIELAB color values [43]. Specifically, pH was slightly more acidic for lighter chicken breasts and slightly more neutral for darker chicken breasts. This may be related to denaturation of myoglobin due to processing [44], however, this effect would be small in the present experiment due to the low amounts of myoglobin present in chicken meat [45]. Unfortunately, the water activity of the processed foods was not measured in the present experiment. Even though it was not anticipated that water activity would be different due to the low inclusion levels of carbohydrate hydrocolloids [1], this information would have enhanced the discussion.

Firmness, toughness, and expressible moisture were affected by the experimental treatments. Specifically, firmness was higher when guar gum was included with another hydrocolloid and toughness increased with higher total carbohydrate hydrocolloid inclusions. Increasing the level of hydrocolloids in a product would increase the gel strength [2]; this is observed in the toughness parameter. Experiments with 0.5–1.5% carbohydrate hydrocolloids in meatballs [6] and restructured hams [4] observed this phenomenon as increased firmness. It may be that 0.5% guar gum, as in DG, in canned pet foods is not enough to influence firmness compared to a sample without a hydrocolloid. It is also possible that the D treatment exhibited enough variability in firmness that the difference compared to DG was not detectable. This is supported by the visually wider spread in the force deformation curves for D vs. all other treatments (Figure S2) and visual inspection of the D treatment cans prior to compression. It was observed that cans filled later in the sequence for that treatment progressively contained more of the solid phase and less of the liquid phase. This likely contributed to the variation in force deformation curves for the D treatment and further illustrated the importance of guar gum keeping ingredients in suspension and evenly distributed. The effect of increased carbohydrate hydrocolloid content was also observed in expressible moisture. As was mentioned in the discussion of consistency, hydrocolloids with hydroxyl groups can form hydrogen bonds with water [2]. Increasing the level of those hydrocolloids introduces more hydroxyl groups, resulting in more bonding with water. This would lower the amount of water that could be expressed and has been observed in restructured hams [4]. The difference between D and DG highlights this well and illustrates the strong power of guar gum to interact with water. It is possible that processing D to a higher total lethality decreased the overall protein functionality [23] and confounded the observed lower toughness and higher level of expressible moisture. In future experiments, treatments with significantly different consistencies could be thermally processed separately to ensure that all treatments receive the same level of processing.

The KCG treatment was firmer and tougher with lower levels of expressible moisture compared to LBG and XGG. This is caused by the different gel structures formed with these hydrocolloids. Gels created by the combination of kappa carrageenan and potassium ions (i.e., from potassium chloride) can withstand substantial application of force before fracturing [46,47]. These gel systems are typically described as “firm” and “brittle” [2]. On the other hand, guar gum, locust bean gum, and xanthan gum form bonds with the hydrogen atoms in water to form a gel structure [2]. Specifically, gels containing xanthan gum and a galactomannan are described as “firm” and “rubbery” [46]. These different gelation mechanisms are defining features in this analysis. For example, a difference in expressible moisture between DG and KCG was not observed or expected because kappa carrageenan does not participate in many hydrogen bonds with water. This concept was echoed in the force deformation curves produced by the texture analysis procedure (Figure S2). The KCG treatment exhibited larger peaks during the deformation vs. the LBG and XGG treatments. As kappa carrageenan gels are “brittle,” the KCG treatment may have fractured multiple times during the compression as increasing levels of force were applied. The LBG and XGG treatments contained gels described as “rubbery” and were able to deform more elastically with less resistance compared to the KCG treatment. As such, the LBG and XGG treatments exhibited smoother force deformation curves compared to the KCG treatment.

Quantitative texture analysis of canned pet food is rarely reported, and expressible moisture has never been documented. As such, the values presented in this manuscript can serve as references for commercial product development and improvement. These metrics may be important to pet owner acceptability and pet palatability and food preference. Reports suggest that cats prefer a softer food requiring less work to chew in the first 7 days of consuming a canned food [28]. The softer textures for LBG and XGG vs. KCG may be preferred by cats, but this was not a focus of the present study. Future work should expand upon this study and utilize palatability testing with dogs and cats as well as consumer testing with pet owners to determine which textures are preferred and why they are preferred.

### 4.4. Proposed Future Research

This work highlighted multiple areas for future research. First, the effect the hydrocolloid concentration has on pre-thermal processing batter consistency, heat penetration, and finished product texture and expressible moisture should be investigated. This would aid in determining the optimal levels of the hydrocolloids evaluated in the present experiment. As learned from this research, dextrose is not a good control ingredient and another should be used to avoid the changes in pH and color that were observed in the present experiment. The use of the primary meat as the control ingredient is standard practice in evaluating the effects of hydrocolloids in restructured meat products for human consumption. Another alternative control ingredient could be cellulose, which is a carbohydrate ingredient but has no effect on viscosity [48]. Second, the Bostwick consistometer should be validated against direct apparent viscosity methods. This could be done simultaneously to other work in an experiment evaluating hydrocolloids. Findings from such an experiment may confirm that Bostwick consistency is an appropriate methodology or suggest that a different method should be the standard. Finally, the changes due to dextrose inclusion at low levels were unexpected. The effect of inclusion level on pH, color, and Maillard reaction products should be explored in the event that dextrose is essential for future experiments or for commercial products.

## 5. Conclusions

Hydrocolloid inclusions affected canned pet foods before, during, and after thermal processing. Thickening batter consistency to 6.60 cm traveled in 30 s or thicker likely decreased the rate of heat penetration and lowered the accumulation of lethality and C_100_. The addition of at least 0.5% guar gum toughened wet pet foods and decreased expressible moisture, but at least 1% hydrocolloid content was needed to observe differences in firmness. Dextrose inclusion at either 0.5% or 1% resulted in lower product pH and increased red and yellow color hues. Replacement of guar gum alone may need to focus on increased consistency prior to thermal processing. On the other hand, researchers should address the greater firmness and toughness and lower expressible moisture observed when kappa carrageenan and guar gum were used in combination compared to guar gum with either xanthan gum or locust bean gum. The differences observed in the present experiment illustrated the importance of hydrocolloids to canned pet foods. These distinctions may influence pet palatability and pet owner preference. Additionally, methodologies for quantifying differences in firmness, toughness, and expressible moisture of canned pet food were described. These methods should be utilized when evaluating new functional and structural ingredient systems for canned pet food.

## Figures and Tables

**Table 1:** Ingredient composition of thermally processed canned pet foods ^1^ containing select hydrocolloids.

Ingredient, % *w*/*w*	D	DG	KCG	LBG	XGG
Mechanically separated chicken	56.00	56.00	56.00	56.00	56.00
Water	38.35	38.35	38.35	38.35	38.35
Brewer’s rice	3.00	3.00	3.00	3.00	3.00
Potassium chloride	0.50	0.50	0.50	0.50	0.50
Spray-dried egg white	0.50	0.50	0.50	0.50	0.50
Sunflower oil	0.50	0.50	0.50	0.50	0.50
Vitamin premix ^2^	0.10	0.10	0.10	0.10	0.10
Trace mineral premix ^3^	0.05	0.05	0.05	0.05	0.05
Dextrose	1.00	0.50	-	-	-
Guar gum	-	0.50	0.50	0.50	0.50
Kappa carrageenan	-	-	0.50	-	-
Locust bean gum	-	-	-	0.50	-
Xanthan gum	-	-	-	-	0.50

^1^ D = 1% dextrose; DG = 0.5% guar gum and 0.5% dextrose; KCG = 0.5% guar gum and 0.5% kappa carrageenan; LBG = 0.5% guar gum and 0.5% locust bean gum; XGG = 0.5% guar gum and 0.5% xanthan gum. ^2^ One kg of vitamin premix supplies 17,163,000 IU vitamin A, 920,000 IU vitamin D3, 79,887 IU vitamin E, 22.0 mg vitamin B12 (cobalamin), 4719 mg vitamin B2 (riboflavin), 12,186 mg vitamin B5 (d-pantothenic acid), 14,252 mg vitamin B1 (thiamin), 64,730 mg vitamin B3 (niacin), 5537 mg vitamin B6 (pyridoxine), 720 mg vitamin B9 (folic acid), and 70.0 mg vitamin B7 (biotin). ^3^ One kg of trace mineral premix supplies 88,000 mg zinc sulfate, 38,910 mg ferrous sulfate, 11,234 mg copper sulfate, 5842 mg manganous oxide, 310 mg sodium selenite, and 1584 mg calcium iodate.

**Table 2:** Batter characteristics of thermally processed canned pet foods ^1^ containing select hydrocolloids.

Measurement	D	DG	KCG	LBG	XGG	SEM	*p*-Value
Consistency, cm/30 s	23.64 ^a^	6.60 ^b^	1.69 ^c^	3.63 ^c^	2.94 ^c^	0.719	<0.0001
pH	5.90	5.93	5.95	5.94	5.97	0.081	0.7411
Water activity	0.984	0.996	0.992	0.987	0.992	0.0067	0.2528

^abc^ Treatment means with unlike superscripts are different (*p* < 0.05). ^1^ D = 1% dextrose; DG = 0.5% guar gum and 0.5% dextrose; KCG = 0.5% guar gum and 0.5% kappa carrageenan; LBG = 0.5% guar gum and 0.5% locust bean gum; XGG = 0.5% guar gum and 0.5% xanthan gum.

**Table 3:** Processing controls of thermally processed canned pet foods ^1^ containing select hydrocolloids.

Measurement	D	DG	KCG	LBG	XGG	SEM	*p*-Value
Number of thermocouples	12	12	11	11	11	-	-
Initial internal can temperature, °C	55.32	56.13	58.51	55.01	53.40	1.768	0.2038
Can fill weight, g	404.6	404.9	405.1	404.3	404.0	0.54	0.3900
Gross headspace, mm	14.83	14.53	13.29	13.17	13.53	0.350	0.4248
Post-processing can vacuum, kPa	−12.4	−11.9	−16.0	−12.7	−11.4	1.76	0.4605

^1^ D = 1% dextrose; DG = 0.5% guar gum and 0.5% dextrose; KCG = 0.5% guar gum and 0.5% kappa carrageenan; LBG = 0.5% guar gum and 0.5% locust bean gum; XGG = 0.5% guar gum and 0.5% xanthan gum.

**Table 4:** Lethality and cook values (C_100_) for thermally processed canned pet foods ^1^ containing select hydrocolloids.

Measurement, min	D	DG	KCG	LBG	XGG	SEM	*p*-Value
Total lethality	20.24 ^a^	18.63 ^b^	18.27 ^b^	18.33 ^b^	18.26 ^b^	0.470	0.0121
Heating lethality	12.08 ^a^	9.71 ^b^	9.25 ^b^	8.74 ^b^	8.66 ^b^	0.566	0.0177
Cooling lethality	8.17 ^b^	8.92 ^ab^	9.01 ^ab^	9.59 ^a^	9.61 ^a^	1.714	0.0428
Total C_100_	201.90	196.41	195.19	196.42	195.56	2.923	0.2307
Heating C_100_	137.01 ^a^	120.67 ^b^	118.84 ^b^	115.38 ^b^	114.06 ^b^	3.870	0.0196
Cooling C_100_	64.90 ^b^	75.74 ^a^	76.36 ^a^	81.04 ^a^	81.50 ^a^	9.233	0.0099

^ab^ Treatment means with unlike superscripts are different (*p* < 0.05). ^1^ D = 1% dextrose; DG = 0.5% guar gum and 0.5% dextrose; KCG = 0.5% guar gum and 0.5% kappa carrageenan; LBG = 0.5% guar gum and 0.5% locust bean gum; XGG = 0.5% guar gum and 0.5% xanthan gum.

**Table 5:** Finished product characteristics of thermally processed canned pet foods ^1^ containing select hydrocolloids.

Measurement	D	DG	KCG	LBG	XGG	SEM	*p*-Value
pH	5.95 ^c^	5.97 ^c^	6.38 ^a^	6.27 ^b^	6.24 ^b^	0.080	<0.0001
Total moisture, %	79.37 ^a^	79.04 ^a^	77.83 ^ab^	77.30 ^b^	77.28 ^b^	0.798	0.0418
EM ^2^, % of sample	49.91 ^a^	26.93 ^b^	23.59 ^b^	15.92 ^c^	17.16 ^c^	1.905	<0.0001
Firmness, N	9.03 ^c^	8.47 ^c^	27.00 ^a^	16.30 ^b^	14.87 ^b^	2.673	<0.0001
Toughness, N·mm	67 ^d^	117 ^c^	370 ^a^	245 ^b^	225 ^b^	32.5	<0.0001
L* ^3^	53.61 ^c^	56.88 ^b^	57.59 ^ab^	59.09 ^a^	58.65 ^ab^	1.044	0.0023
a* ^4^	8.18 ^a^	8.56 ^a^	4.03 ^b^	4.68 ^b^	4.51 ^b^	1.244	0.0108
b* ^5^	21.40 ^a^	22.69 ^a^	14.64 ^b^	15.93 ^b^	15.59 ^b^	1.511	<0.0001

^abcd^ Treatment means with unlike superscripts are different (*p* < 0.05). ^1^ D = 1% dextrose; DG = 0.5% guar gum and 0.5% dextrose; KCG = 0.5% guar gum and 0.5% kappa carrageenan; LBG = 0.5% guar gum and 0.5% locust bean gum; XGG = 0.5% guar gum and 0.5% xanthan gum. ^2^ EM = expressible moisture. ^3^ L* represents the lightness/darkness scale of color; values closer to 100 indicate lighter products and values closer to zero indicate darker products. ^4^ a* represents the red/green scale of color; more positive values indicate redder colors and more negative values indicate greener colors. ^5^ b* represents the yellow/blue scale of color; more positive values indicate yellower colors and more negative values indicate bluer colors.

## Data Availability

The data presented in this study are available upon request from the corresponding author.

## Supplementary Materials

The following are available online at https://www.mdpi.com/article/10.3390/foods10102506/s1, Figure S1: Internal can temperatures for thermally processed wet pet foods containing different carbohydrate hydrocolloid ingredients ^1^; Figure S2: Force deformation curves from modified back extrusion procedure applied to thermally processed wet pet foods containing different carbohydrate hydrocolloid ingredients ^1^.
