# Supplementary material for: The Effects of Select Hydrocolloids on the Processing of Pâté-Style Canned Pet Food
Source: Foods. 2021 Oct 19;10(10):2506. doi: 10.3390/foods10102506 (PMC8535922; doi:10.3390/foods10102506)
Supplement: Supplementary file 1 [file foods-10-02506-s001.zip › foods-1403321-supplementary.pdf]

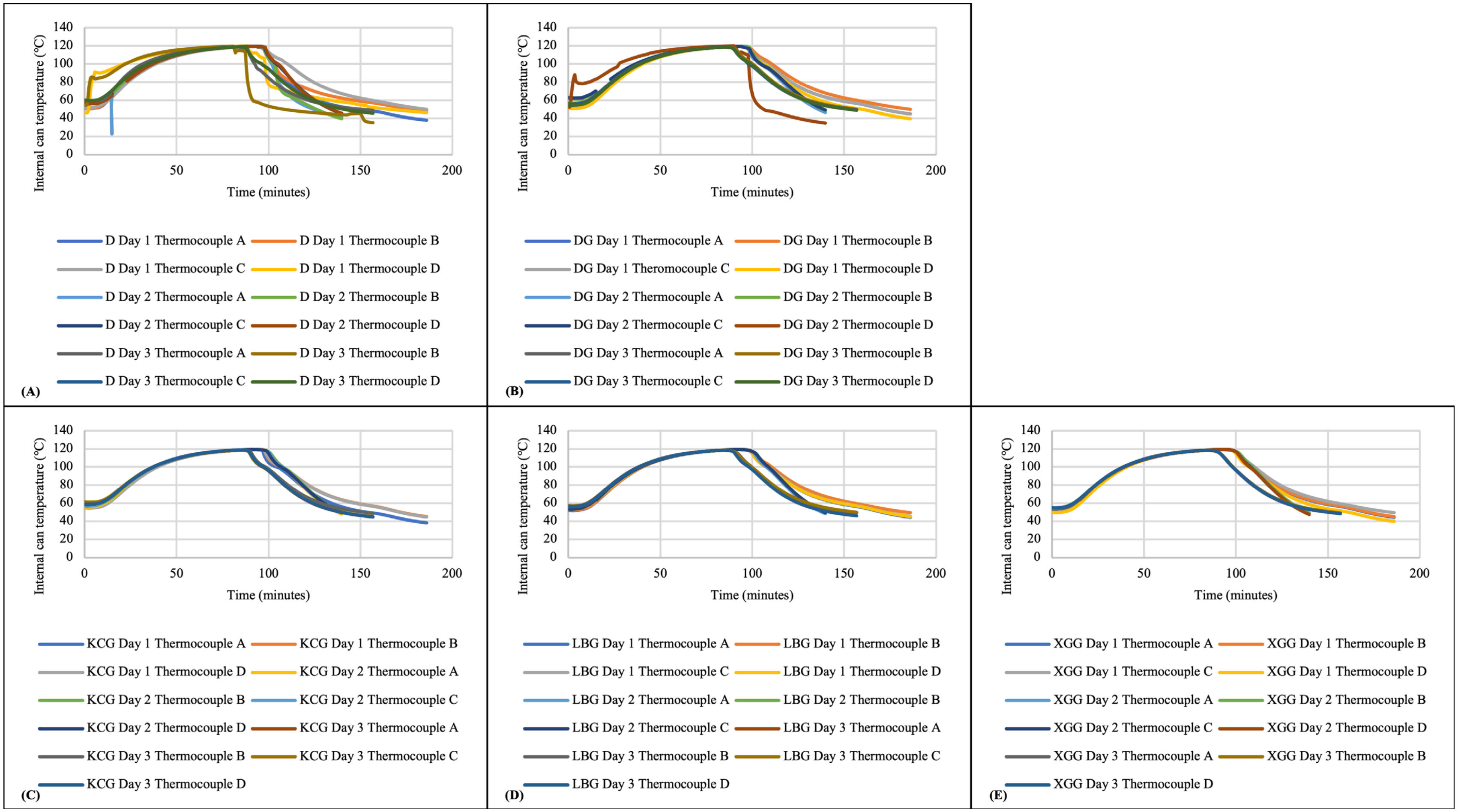


**Figure S1: Internal can temperatures during thermal processing for canned pet foods containing select hydrocolloids [1].**

[1] D = 1% dextrose; DG = 0.5% guar gum and 0.5% dextrose; KCG = 0.5% guar gum and 0.5% kappa carrageenan; LBG = 0.5% guar gum and 0.5% locust bean gum; XGG = 0.5% guar gum and 0.5% xanthan gum.

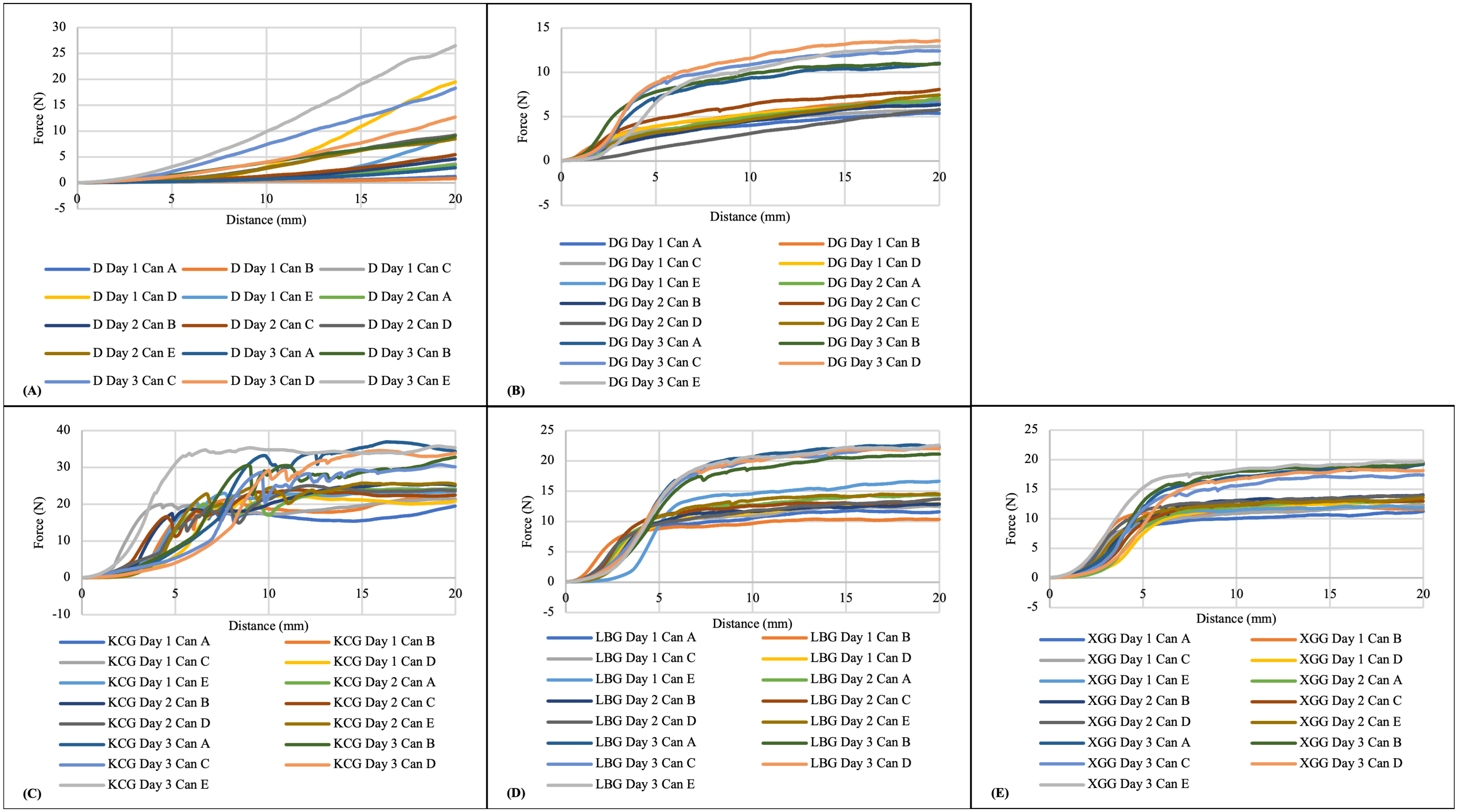


**Figure S2: Force deformation curves from modified back extrusion procedure applied to thermally processed canned pet foods containing select hydrocolloids[1].**

[1] D = 1 % dextrose; DG = 0.5% guar gum and 0.5% dextrose; KCG = 0.5% guar gum and 0.5% kappa carrageenan; LBG = 0.5% guar gum and 0.5% locust bean gum; XGG = 0.5% guar gum and 0.5% xanthan gum.
